# Association of Hypertensive Intracerebral Hemorrhage with Left Ventricular Hypertrophy on Transthoracic Echocardiography

**DOI:** 10.3390/jcm9072148

**Published:** 2020-07-08

**Authors:** Lars-Peder Pallesen, Jenny Wagner, Dimitris Lambrou, Silke Braun, Matthias Weise, Alexandra Prakapenia, Jessica Barlinn, Timo Siepmann, Simon Winzer, Haidar Moustafa, Hagen H. Kitzler, Kristian Barlinn, Heinz Reichmann, Volker Puetz

**Affiliations:** 1 Dresden Neurovascular Center, Department of Neurology, Dresden University of Technology, 01307 Dresden, Germany; jty_89@web.de (J.W.); alexandra.prakapenia@uniklinikum-dresden.de (A.P.); jessica.barlinn@uniklinikum-dresden.de (J.B.); timo.siepmann@uniklinikum-dresden.de (T.S.); simon.winzer@uniklinikum-dresden.de (S.W.); haidar.moustafa@uniklinikum-dresden.de (H.M.); kristian.barlinn@uniklinikum-dresden.de (K.B.); heinz.reichmann@uniklinikum-dresden.de (H.R.); volker.puetz@uniklinikum-dresden.de (V.P.); 2 Department of Medicine, University of Thessaly, 41500 Larissa, Greece; dnlambrou@gmail.com; 3 Medical Clinic I, Dresden University of Technology, 01307 Dresden, Germany; silke.braun@uniklinikum-dresden.de; 4 Medical Clinic III, Dresden University of Technology, 01307 Dresden, Germany; matthias.weise@uniklinikum-dresden.de; 5 Institute of Diagnostic and Interventional Neuroradiology, Dresden University of Technology, 01307 Dresden, Germany; hagen.kitzler@uniklinikum-dresden.de

**Keywords:** intracerebral hemorrhage, hypertensive cardiomyopathy, left ventricular hypertrophy, arterial hypertension, echocardiography

## Abstract

Introduction: Arterial hypertension is the most frequent cause for spontaneous intracerebral hemorrhage (sICH) and may also cause left ventricular hypertrophy (LVH). We sought to analyze whether hypertensive sICH etiology is associated with LVH. Methods: We analyzed consecutive patients with sICH who were admitted to our tertiary stroke center during a four-year period and underwent transthoracic echocardiography (TTE) as part of the diagnostic work-up. We defined hypertensive sICH as typical localization of hemorrhage in patients with arterial hypertension and no other identified sICH etiology. We defined an increased end-diastolic interventricular septal wall thickness of ≥11 mm on TTE as a surrogate parameter for LVH. Results: Among 395 patients with sICH, 260 patients (65.8%) received TTE as part of their diagnostic work-up. The median age was 71 years (interquartile range (IQR) 17), 160 patients (61.5%) were male, the median baseline National Institute of Health Stroke Scale (NIHSS) score was 8 (IQR 13). Of these, 159 (61.2%) patients had a hypertensive sICH and 156 patients (60%) had LVH. In univariable (113/159 (71.1%) vs. 43/101 (42.6%); odds ratio (OR) 3.31; 95% confidence interval (CI_95%_) 1.97–5.62); and multivariable (adjusted OR 2.95; CI_95%_ 1.29–6.74) analysis, hypertensive sICH was associated with LVH. Conclusions: In patients with sICH, LVH is associated with hypertensive bleeding etiology. Performing TTE is meaningful for diagnosis of comorbidities and clarification of bleeding etiology in these patients. Future studies should include long-term outcome parameters and assess left ventricular mass as main indicator for LVH.

## 1. Introduction

Stroke is the second leading cause of death worldwide and the leading cause for acquired permanent disability in adults [1]. Among etiologies, hemorrhagic stroke accounts for approximately 15% of patients [2,3]. Spontaneous intracerebral hemorrhage (sICH) is associated with a high risk of short-term mortality and long-term functional dependence [3,4]. The most frequent pathophysiological mechanism causing sICH is chronic arterial hypertension with development and eventual rupture of microscopic pseudoaneurysms of penetrating arteries typically localized in the basal ganglia, thalami, pons, midbrain and cerebellum [5,6,7].

Left ventricular hypertrophy (LVH) is another frequent complication of long-term arterial hypertension and is usually attributed as an adaption to an increased afterload [8]. It is an independent predictor for sudden cardiac death, ventricular arrhythmias, coronary artery disease and heart failure [9,10]. Transthoracic echocardiography (TTE) is the primary non-invasive modality for the assessment of cardiac structure and function, including the determination of LVH [8,11]. Whilst the diagnostic yield of transthoracic and transesophageal echocardiography in ischemic stroke has been studied extensively, there is only limited data regarding the diagnostic value of TTE in patients with sICH, especially regarding the detection of LVH as a significant comorbidity [12].

We tested the hypothesis whether hypertensive sICH etiology is associated with LVH in routine TTE examinations.

## 2. Methods

### 2.1. Patients

We performed a retrospective, single-center, observational cohort study. We analyzed consecutive adult patients with sICH who were admitted to our tertiary care stroke center from 01/2010 to 12/2013 and had a TTE as part of their diagnostic work-up. TTE was routinely performed in patients with sICH in our center during the study period. Data acquisition was performed via electronic chart review of our Clinical Information System (ORBIS^®^, AGFA Healthcare, Mortsel, Belgium) including electronic discharge summaries and TTE image files of individual patients. Recorded data included clinical baseline characteristics, cardiovascular risk factors, National Institute of Health Stroke Scale (NIHSS) score on admission and modified Rankin Scale (mRS) at discharge. We dichotomized the discharge mRS score into favorable outcome (mRS scores 0–3) and unfavorable outcome (mRS scores 4–6). Furthermore, we used a Sokolow–Lyon Index ≥ 35 mV as an electrocardiogram (ECG) criterion for LVH. We defined arterial hypertension either as known pre-existing arterial hypertension or by diagnosis of the treating stroke neurologist during the hospital course according to European guidelines [13].

Brain imaging was primarily performed with non-contrast computed tomography scan (CT) on admission. We defined hypertensive sICH as intracerebral hemorrhage in hypertensive patients with typical localization (i.e., basal ganglia, thalami, pons, midbrain and cerebellum) and without other potential sICH etiologies (e.g., cavernoma, arteriovernous malformation, tumor) [5]. Further sICH etiologies were classified based on results of CT-angiography (CTA), magnetic resonance imaging (MRI) and digital subtraction angiography (DSA) as indicated by the treating stroke neurologist and neuroradiologist.

The local institutional ethics committee (IRB No EK 422112014) approved the study protocol and waived the need for informed consent due to the retrospective and non-interventional nature of our analysis.

### 2.2. Echocardiographic Findings

The ultrasound systems Philips IE33 mi6 (Philips, Amsterdam, Netherlands) was used for two-dimensional TTE in all patients. The TTE examinations were performed according to the guidelines of the German Society of Cardiology, which are comparable to current international and European guidelines [8,14]. We recorded left ventricular diameter, valve functions, left ventricular ejection fraction and diastolic dysfunction. We defined an end-diastolic interventricular septal wall thickness (IVSTd) ≥11 mm as a surrogate parameter for LVH. We categorized the extent of relevant increase of IVSTd as mild (11–13 mm), moderate (14–16 mm) or severe (>16 mm) [15]. Diastolic dysfunction was estimated by the ratio between early mitral inflow velocity and mitral annular early diastolic velocity (E/e’ > 14) in the apical 4-chamber view [16]. Echocardiographic measurements were usually reported based on three representative cycles. An experienced cardiologist retrospectively analyzed all TTE reports and electronically stored images.

### 2.3. Statistical Analysis

Data are reported using standard descriptive statistics. Statistical summaries are displayed using count (percentages) for categorical variables and median with interquartile range (IQR) for continuous outcomes. Univariable and multivariable logistic regression analysis was employed to assess the association of sICH with the pre-selected covariates including LVH. If estimation problems were encountered between the response and a covariate during the univariable analysis, then this covariate was excluded from further consideration in the multivariable analysis (see Supplementary Materials Table S1). In the multivariable analysis, imputation of missing values was carried out using a multiple chain equations methodology. This methodology allows the generation of five complete datasets, followed by analysis of each dataset separately. Backward elimination techniques were used to select important covariates in each complete dataset analysis. The final outcome was produced by appropriately combining the results of the five imputed analyses. In all analyses, a type I error rate of 5% was used (e.g., *p* < 0.05). The R package (R version 3.6.2) was used throughout.

## 3. Results

### 3.1. Patients

**Figure 1:**
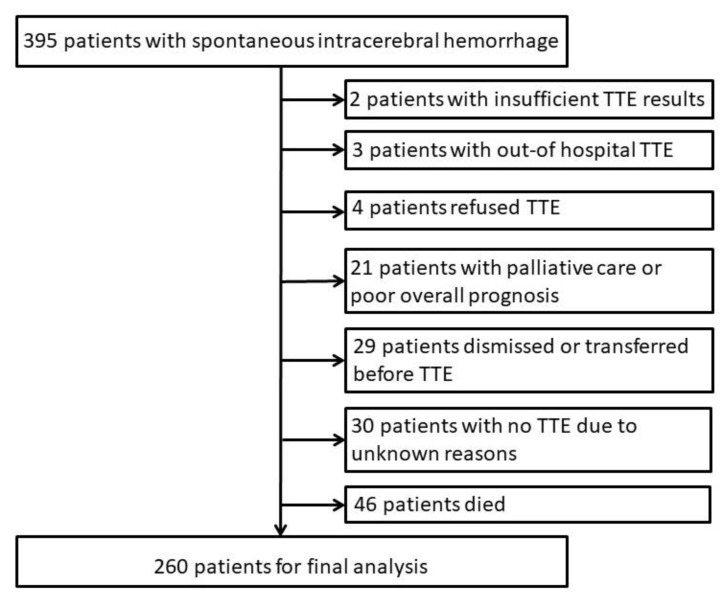
Study flow diagram; TTE indicates transthoracic echocardiography.

During the study period, 395 patients with sICH were admitted to our stroke center. Of these, 260 (65.8%) patients received TTE as part of their work-up. Main reason not to perform TTE was death during the hospital course in 46 (34%) patients (Figure 1). In 30 patients (22.2%), the reason for the cancellation of TTE could not be determined retrospectively.

Of the remaining 260 patients, 160 (61.5%) patients were male, the median age was 71 years (interquartile range (IQR) 17) and the median baseline NIHSS score was 8 (IQR 13). The median systolic blood pressure on admission was 165 mmHg (IQR 31.2). Further baseline characteristics are summarized in Table 1 and Supplementary Materials Table S1). All patients received a non-contrast CT as baseline imaging with 22 (8.5%) patients receiving additional CTA, 128 (49.2%) patients receiving additional MRI and 41 (15.8%) patients receiving additional DSA. Regarding etiology of sICH, 159 patients (61.2%) were classified as having hypertensive sICH. Further sICH etiologies were cerebral amyloidangiopathy in 22 (8.5%) patients, arteriovenous malformation in 13 (5.0%) patients and sinus thrombosis in 2 (0.8%) patients. In 64 (24.6%) patients, no clear sICH etiology could be determined.

Compared to patients with non-hypertensive sICH, hypertensive sICH patients had higher systolic blood pressure on admission (median 169 mmHg (IQR 35) vs. 160 mmHg (IQR 32.5); odds ratio (OR) 1.01; 95% confidence interval (CI_95%_) 1.00–1.02), were more frequently diabetic (45/159 (28.3%) vs. 17/101 (16.8%); OR 1.95, CI_95%_ 1.06–3.72) and had less frequently hyperlipidemia (72/159 (45.3%) vs. 59/101 (58.4%), OR 0.59, CI_95%_ 0.35–0.97). Furthermore, patients with hypertensive sICH had more frequently an unfavorable functional outcome at discharge (96/156 (61.5%) vs. 43/98 (43.9%); OR 2.05; CI_95%_ 1.23–3.43).

### 3.2. Transthoracic Echocardiography (TTE) Findings

Regarding the TTE results of the whole study population, 156 (60%) patients had LVH which was considered as mild in 107 patients (41.2%), moderate in 35 patients (13.5%) and severe in 14 patients (5.4%). Other major findings on TTE were diastolic dysfunction in 214 patients (82.3%) and mitral valve regurgitation in 139 patients (53.5%) (Table 2 and Supplementary Materials Table S1).

### 3.3. Association of TTE Findings with Spontaneous Intracerebral Hemorrhage (sICH) Etiology

Patients with hypertensive sICH had more frequently LVH compared to patients with non-hypertensive sICH etiology (113/159 (71.1%) vs. 43/101 (42.6%); OR 3.31, CI_95%_ 1.97–5.62). This finding was consistent for all LVH categories, i.e., mild (79/159 (49.7%) vs. 28/101 (27.7%); OR 3.56, CI_95%_ 2.01–6.42), moderate (23/159 (14.5%) vs. 12/101 (11.9%); OR 2.42, CI_95%_ 1.10–5.51) and severe LVH (11/159 (6.9%) vs. 3/101 (3.0%), OR 4.62, CI_95%_ 1.35–21.33). Furthermore, an increased left atrial diameter was more frequently diagnosed in patients with hypertensive sICH (41 mm (IQR 8) vs. 38 mm (IQR 9); OR 1.04; CI_95%_ 1.00–1.09; and 82/157 (52.2%) vs. 37/100 (37%); OR 1.86; CI_95%_ 1.12–3.13).

In multivariable analysis, hypertensive sICH was associated with LVH (OR 2.95; CI_95%_ 1.29–6.74). Further factors associated with sICH compared to non-hypertensive sICH etiologies are summarized in Table 3.

## 4. Discussion

Our study demonstrates that hypertensive sICH is associated with LVH as estimated by increased IVSTd on TTE. Additional pathological findings on TTE such as diastolic dysfunction and mitral valve regurgitation were present in more than half of our study population, underlining the diagnostic value of TTE in patients with sICH.

Whilst the diagnostic yield of echocardiography is well examined in patients with ischemic stroke, there is considerable lack of data in patients with sICH [12,17,18]. However, TTE may be helpful in patients with sICH for fluid management, estimating candidacy for neurosurgery and in the selection of specific antihypertensive agents [12]. The available publications suffer from small study populations, missing definition of LVH and sICH etiology or focus on certain risk groups (i.e., patients with cocaine abuse vs. patients without cocaine abuse) [19,20,21]. Furthermore, none of these studies has analyzed the association of LVH with sICH etiology as an important complication of arterial hypertension.

Almost all patients in our study had arterial hypertension, making the sheer occurrence of an elevated blood pressure unsuitable for the diagnosis of hypertensive sICH or LVH. Elevated blood pressure might be the reason for sICH in the first place, a contributing factor or caused by sICH itself (e.g., due to pain, anxiety or sympathetic activation) [7,22]. A previous analysis of 251 patients with sICH suggested that pre-existing arterial hypertension is associated with higher median blood pressure on admission and LVH [22]. However, it is noteworthy that only 29% of the patients received TTE and the authors did not compare LVH with sICH etiology. In our analysis, sICH was associated with LVH even after the adjustment for further co-variates including arterial hypertension and systolic blood pressure on admission, thus underlining its pathophysiological and diagnostic relevance.

Of note, our analysis demonstrated a significant association of hypertensive sICH etiology with LVH across all LVH severity grades, underlining the connection of hypertensive bleeding etiology with possible hypertensive heart disease. The larger left atrial diameter in the hypertensive sICH group supports this conclusion as it is frequently observed in patients with long-standing arterial hypertension [23,24].

Although there is still uncertainty concerning blood pressure control in patients with acute sICH, current evidence and guidelines recommend achieving values below 140 mmHg [25,26]. This is also demonstrated in our data with considerably lower blood pressure 24 h after admission (see Supplementary Materials Table S1), though we cannot comment whether this was caused by drug treatment or spontaneous drop of blood pressure after the hyperacute phase.

The remodeling of the heart due to long term arterial hypertension is a complex process and is not limited to pure anatomical changes [9,27]. Although most research focused on echocardiographic changes in the form of LVH due to hypertensive heart disease, structural and functional adaptions including cardiac fibrosis and alterations of individual cardiac chambers and the arterial system are of rising interest [27]. However, since the diagnosis of cellular changes may require endomyocardial biopsy, TTE as a non-invasive and relatively inexpensive diagnostic method remains of paramount importance in the assessment of myocardial changes due to arterial hypertension [8,9,28,29]. Particularly given the fact that antihypertensive therapy leading to a regression of LVH can improve the patients’ cardiovascular outcomes [30,31].

Of growing importance as a complementary modality of imaging is cardiovascular magnetic resonance (CMR), a method in which not only the cardiac mass, but also interstitial myocardial fibrosis can be detected, which may be helpful to distinguish hypertensive heart disease from other cardiomyopathies [32].

The most precise and, in guidelines, recommended echocardiographic measurement for LVH is the determination of left ventricular mass (LVM) [11,33]. In a large observational cohort study with 2545 patients, the measurement of LVM (adjusted for sex and body surface area) was superior in the detection of significant LVH and provided a more sensitive risk marker for death compared to sole septal thickness cut-offs [33]. Since body weight and height were not consistently recorded during the study period, we used IVSTd as a surrogate marker for LVH. Comparative studies of LVH and IVSTd show an acceptable agreement between both techniques, and measurement of septal wall thickness is relatively easy to determine as part of routine TTE examination even in critically ill patients, is frequently used in clinical practice and its association with arterial hypertension and LVH is well established [15,33]. Nevertheless, considering the operator dependence of IVSTd measurement and the fact that it is not normalized to body surface area, future studies should include measurement of LVM as a marker for LVH. Furthermore, apart from the sole increase of cardiac mass, changes of left ventricular geometry including relative wall thickness may also be caused by arterial hypertension, although this association is not as uncontested as LVH [9].

The most frequent pathological TTE finding of our study was diastolic dysfunction, a condition that constitutes an insufficient filling of the left ventricle during the diastole due to impaired left ventricular relaxation and increased left ventricular stiffness [16,34]. Risk factors for diastolic dysfunction are age, arterial hypertension, LVH and diabetes mellitus [34]. The echocardiographic proof of diastolic dysfunction is considered as one of the criteria for the diagnosis of heart failure with preserved ejection fraction (HFpEF) [35]. A recent meta-analysis supported a multivariable- based echocardiographic approach to determine diastolic dysfunction and demonstrated a potential additional diagnostic value of exercise echocardiography [36].

In our analysis, hypertensive sICH was associated with LVH also after adjustment for the recorded risk factors. However, we cannot exclude that other heart conditions like diabetic cardiomyopathy, which can also present with diastolic relaxation abnormalities, LVH and left atrial enlargement, might have influenced our results [37].

Our study has limitations. We cannot comment on the impact of LVH or further TTE findings on long-term functional outcomes of our patients. Future analysis should include long-term parameters, for example mRS at three and 12 months as well as repeated TTE examinations. Moreover, we cannot exclude that some patients with non-hypertensive sICH had a hypertensive sICH etiology and vice versa. This may partly explain the high prevalence of LVH in patients with non-hypertensive sICH etiologies in our study. However, this would further support the recommendation to establish TTE as part of the diagnostic work-up in all patients with sICH regardless of ICH etiology. Furthermore, we cannot provide data on quality of blood pressure control before admission or after discharge of the patients. Finally, as end diastolic posterior wall thickness was not consistently reported in the TTE report during the study period, we are also unable to comment on non body-surface-area normalized LVM.

## 5. Conclusions

In patients with sICH, a hypertensive sICH etiology was associated with LVH as diagnosed by measurement of IVSTd on TTE. Moreover, the majority of patients had further clinically relevant echocardiographic findings. Our study underlines that performing a TTE is clinically meaningful for the diagnosis of comorbidities and clarification of bleeding etiology in these patients. Therefore, physicians dealing with acute care of patients with sICH should consider performing TTE as part of the routine work-up. Furthermore, our analysis might be the foundation for further research, preferably in prospective settings with long-term neurological outcomes and repeated TTE controls. Future studies should be performed according to current guidelines, especially with the inclusion of body surface area-normalized LVM as a more precise parameter for LVH.

## Figures and Tables

**Table 1:** Selected baseline characteristics of the whole study population and comparison of patients with and without hypertensive spontaneous intracerebral hemorrhage (sICH); a complete table of all variables can be found in Supplementary Materials Table S1. ECG indicates electrocardiogram.

	Study Population (*N* = 260)	Hypertensive sICH (*N* = 159)	Non-Hypertensive sICH (*N* = 101)	OR	CI_95%_
Age, median (IQR)	71.0 (17.0)	71.0 (18.5)	72.0 (11.0)	0.98	0.96–1.01
Sex, male, n/N (%)	160/260 (61.5)	102/159 (64.2)	58 /101 (57.4)	1.33	0.80–2.21
NIHSS on admission, median (IQR)	8.0 (13.0)	9.0 (11.0)	7.0 (14.0)	1.01	0.99–1.04
Systolic blood pressure on admission, mmHg, median (IQR)	165.0 (31.2)	169.0 (35.0)	160.0 (32.5)	1.01	1.00–1.02 *
Pre-existing ischemic stroke, n/N (%)	60/260 (23.1)	32/159 (20.1)	28/101 (27.7)	0.66	0.37–1.18
Arterial hypertension, n/N (%)	257/260 (98.8)	159/159 (100.0)	98/101 (97.0)	NA	NA–NA
Hyperlipidemia, n/N (%)	131/260 (50.4)	72/159 (45.3)	59/101 (58.4)	0.59	0.35–0.97 *
Diabetes, n/N (%)	62/260 (23.8)	45/159 (28.3)	17/101 (16.8)	1.95	1.06–3.72 *
Peripheral vascular disease, n/N (%)	12/260 (4.6)	9/159 (5.7)	3/101 (3.0)	1.96	0.57–9.00
Coronary artery disease, n/N (%)	36/260 (13.8)	21/159 (13.2)	15/101 (14.9)	0.87	0.43–1.81
Smoking, n/N (%)	37/260 (14.2)	23/159 (14.5)	14/101 (13.9)	1.05	0.52–2.20
Atrial fibrillation, n/N (%)	22/260 (8.5)	11/159 (6.9)	11/101 (10.9)	0.61	0.25–1.48
Antihypertensive drugs on admission					
one, n/N (%)	53/224 (23.7)	27/137 (19.7)	26/87 (29.9)	0.55	0.27–1.12
more than one, n/N (%)	93/224 (41.5)	59/137 (43.1)	34/87 (39.1)	0.92	0.49–1.72
Hypertrophy on ECG, n/N (%)	15/245 (6.1)	8/149 (5.4)	7/96 (7.3)	0.72	0.25–2.12
Aspirin on admission, n/N (%)	60/246 (24.4)	32/151 (21.2)	28/95 (29.5)	0.73	0.40–1.35
Anticoagulation on admission, n/N (%)	36/246 (14.6)	28/151 (18.5)	8/95 (8.4)	2.24	0.99–5.58
Treatment on ICU, n/N (%)	83/260 (31.9)	49/159 (30.8)	34/101 (33.7)	0.88	0.52–1.50
Surgery, n/N (%)	33/260 (12.7)	21/159 (13.2)	12/101 (11.9)	1.13	0.54–2.47
Intraventricular bleeding, n/N (%)	78/260 (30.0)	51/159 (32.1)	27/101 (26.7)	1.29	0.75–2.27
Length of hospital treatment, days, median (IQR)	11.0 (8.0)	11.0 (9.0)	11.0 (6.0)	1.03	0.99–1.07
Length of ICU treatment, days, median (IQR)	5.0 (11.0)	5.0 (11.0)	5.0 (10.0)	1.02	0.99–1.06
Discharge to rehabilitation, n/N (%)	235/260 (90.4)	146/159 (91.8)	89/101 (88.1)	1.51	0.65–3.48
mRS 4-6 at discharge, n/N (%)	139/254 (54.7)	96/156 (61.5)	43/98 (43.9)	2.05	1.23–3.43 *

ICU, intensive care unit; IQR, interquartile range; mRS, modified Rankin Scale; NIHSS, National Institute of Health Stroke Scale; * indicates variables with significant association; OR, odds ratio; CI_95%_, 95% confidence interval.

**Table 2:** Comparison of TTE results in patients with and without hypertensive spontaneous intracerebral hemorrhage (sICH).

	Hypertensive sICH	Non-Hypertensive sICH	OR	CI_95%_
End diastolic interventricular septum wall thickness, mm, median (IQR)	13.0 (2.0)	12.0 (2.0)	1.35	1.17–1.58 *
Left ventricular hypertrophy				
any, n/N (%)	113/159 (71.1)	43/101 (42.6)	3.31	1.97–5.62 *
mild, n/N (%)	79/159 (49.7)	28/101 (27.7)	3.56	2.01–6.42 *
moderate, n/N (%)	23/159 (14.5)	12/101 (11.9)	2.42	1.10–5.51 *
severe, n/N (%)	11/159 (6.9)	3/101 (3.0)	4.62	1.35–21.33 *
Left atrial enlargement, n/N (%)	82/157 (52.2)	37/100 (37.0)	1.86	1.12–3.13 *
Diastolic dysfunction, n/N (%)	132/159 (83.0)	82/101 (81.2)	1.13	0.59–2.16
Mitral valve regurgitation, n/N (%)	81/159 (50.9)	58/101 (57.4)	0.77	0.46–1.27
Tricuspid valve regurgitation, n/N (%)	67/159 (42.1)	50/100 (50.0)	0.73	0.44–1.20
Aortic valve regurgitation, n/N (%)	36/159 (22.6)	29/100 (29.0)	0.72	0.41–1.27
Aortic valve stenosis, n/N (%)	8/159 (5.0)	10/101 (9.9)	0.48	0.18–1.27
Diameter left atrium, mm, median (IQR)	41.0 (8.0)	38.0 (9.0)	1.04	1.00–1.09 *
Left ventricular end-diastolic diameter, mm, median (IQR)	46.0 (8.0)	44.0 (7.0)	1.02	0.98–1.06
Left ventricular ejection fraction, per cent, median (IQR)	60.0 (5.0)	60.0 (0.0)	0.99	0.95–1.04
Right ventricular ejection fraction, per cent, median (IQR)	60.0 (5.0)	60.0 (0.0)	1.00	0.95–1.07

IQR indicates interquartile range, OR, odds ratio; * indicates significant association; CI_95%_, 95% confidence interval.

**Table 3:** Multivariable analysis for the association of hypertensive sICH with clinical and echocardiographic parameters.

	OR	CI_95%_
LVH	2.95	1.29–6.74
NIHSS on admission	0.96	0.92–1.00
Systolic blood pressure on admission	1.04	1.01–1.07
Hyperlipidemia	0.46	0.21–0.99
Pre-existing ischemic stroke	0.41	0.18–0.95
Atrial fibrillation	0.26	0.07–0.98
Left atrial enlargement	3.33	1.39–7.99
Diastolic dysfunction	0.28	0.09–0.82

OR indicates odds ratio; LVH, left ventricular hypertrophy; NIHSS, National Institute of Health Stroke Scale.

## Supplementary Materials

The following are available online at https://www.mdpi.com/2077-0383/9/7/2148/s1: Table S1: List of all variables included in our analysis.
