# Supplementary material for: Association of Hypertensive Intracerebral Hemorrhage with Left Ventricular Hypertrophy on Transthoracic Echocardiography
Source: J Clin Med. 2020 Jul 8;9(7):2148. doi: 10.3390/jcm9072148 (PMC7408960; doi:10.3390/jcm9072148)
Supplement: Supplementary file 1 [file jcm-09-02148-s001.docx]

**Supplemental Table S1:** List of all variables included in our analysis; ICU indicates intensive care unit; IQR, interquartile range; OR, Odds ratio; mRS, modified Rankin Scale; NIHSS, National Institute of Health Stroke Scale,† variable had to be excluded from the multivariable analysis due to estimation problems.

| Variable | Study Population  (n=260) | Hypertensive sICH  (n=159) | Non-hypertensive sICH  (n=101) | OR | CI_95%_ |
| --- | --- | --- | --- | --- | --- |
| Age, median (IQR) | 71.0 (17.0) | 71.0 (18.5) | 72.0 (11.0) | 0.98 | 0.96 - 1.01 |
| Sex, male, n/N (%) | 160 / 260 (61.5) | 102 / 159 (64.2) | 58 / 101 (57.4) | 1.33 | 0.80 - 2.21 |
| NIHSS on admission, median (IQR) | 8.0 (13.0) | 9.0 (11.0) | 7.0 (14.0) | 1.01 | 0.99 - 1.04 |
| mRS on admission (4-6), n/N (%) | 167 / 254 (65.7) | 115 / 156 (73.7) | 52 / 98 (53.1) | 2.48 | 1.46 - 4.25 |
| mRS 4-6 on discharge, n/N (%) | 139 / 254 (54.7) | 96 / 156 (61.5) | 43 / 98 (43.9) | 2.05 | 1.23 - 3.43 |
| Systolic blood pressure on admission, median (IQR) | 165.0 (31.2) | 169.0 (35.0) | 160.0 (32.5) | 1.01 | 1.00 - 1.02 |
| Diastolic blood pressure on admission, median (IQR) | 82.5 (22.8) | 86.5 (23.2) | 80.0 (20.0) | 1.03 | 1.01 - 1.05 |
| Systolic blood pressure 24hrs after admission, median (IQR) | 141.0 (25.0) | 143.0 (25.0) | 140.0 (21.5) | 1.00 | 0.99 - 1.02 |
| Diastolic blood pressure 24hrs after admission, median (IQR) | 70.0 (18.0) | 70.0 (17.2) | 70.0 (18.2) | 1.01 | 0.99 - 1.03 |
| Heart frequency on admission, median (IQR) | 77.0 (23.5) | 78.0 (23.0) | 74.0 (20.0) | 1.01 | 1.00 - 1.03 |
| Heart frequency 24hrs after admission, median (IQR) | 73.0 (21.0) | 72.5 (25.5) | 74.0 (15.0) | 1.00 | 0.99 - 1.02 |
| Pre-existing ischemic stroke, n/N (%) | 60 / 260 (23.1) | 32 / 159 (20.1) | 28 / 101 (27.7) | 0.66 | 0.37 - 1.18 |
| Arterial hypertension†, n/N (%) | 257 / 260 (98.8) | 159 / 159 (100.0) | 98 / 101 (97.0) | NA | NA - NA |
| Hyperlipidemia, n/N (%) | 131 / 260 (50.4) | 72 / 159 (45.3) | 59 / 101 (58.4) | 0.59 | 0.35 - 0.97 |
| Diabetes, n/N (%) | 62 / 260 (23.8) | 45 / 159 (28.3) | 17 / 101 (16.8) | 1.95 | 1.06 - 3.72 |
| Peripheral vascular disease, n/N (%) | 12 / 260 (4.6) | 9 / 159 (5.7) | 3 / 101 (3.0) | 1.96 | 0.57 - 9.00 |
| Coronary artery disease, n/N (%) | 36 / 260 (13.8) | 21 / 159 (13.2) | 15 / 101 (14.9) | 0.87 | 0.43 - 1.81 |
| Smoking, n/N (%) | 37 / 260 (14.2) | 23 / 159 (14.5) | 14 / 101 (13.9) | 1.05 | 0.52 - 2.20 |
| Antihypertensive drugs on admission |  |  |  |  |  |
| none, n/N (%) | 78 / 224 (34.8) | 51 / 137 (37.2) | 27 / 87 (31.0) |  |  |
| one, n/N (%) | 53 / 224 (23.7) | 27 / 137 (19.7) | 26 / 87 (29.9) | 0.55 | 0.27 - 1.12 |
| more than one, n/N (%) | 93 / 224 (41.5) | 59 / 137 (43.1) | 34 / 87 (39.1) | 0.92 | 0.49 - 1.72 |
| any, n/N (%) | 146 / 224 (65.2) | 86 / 137 (62.8) | 60 / 87 (69.0) | 0.76 | 0.43 - 1.34 |
| Hypertrophy on ECG, n/N (%) | 15 / 245 (6.1) | 8 / 149 (5.4) | 7 / 96 (7.3) | 0.72 | 0.25 - 2.12 |
| No antiplatelet agent or anticoagulant on admission, n/N (%) | 146 / 246 (59.3) | 89 / 151 (58.9) | 57 / 95 (60.0) |  |  |
| Aspirin on admission, n/N (%) | 60 / 246 (24.4) | 32 / 151 (21.2) | 28 / 95 (29.5) | 0.73 | 0.40 - 1.35 |
| Anticoagulant on admission, n/N (%) | 36 / 246 (14.6) | 28 / 151 (18.5) | 8 / 95 (8.4) | 2.24 | 0.99 - 5.58 |
| Anticoagulation other than Aspirin or anticoagulant, n/N (%) | 4 / 246 (1.6) | 2 / 151 (1.3) | 2 / 95 (2.1) | 0.64 | 0.08 - 5.46 |
| Any anticoagulant or antiplatelet agent on admission, n/N (%) | 100 / 246 (40.7) | 62 / 151 (41.1) | 38 / 95 (40.0) | 1.04 | 0.62 - 1.77 |
| Treatment on ICU, n/N (%) | 83 / 260 (31.9) | 49 / 159 (30.8) | 34 / 101 (33.7) | 0.88 | 0.52 - 1.50 |
| Surgery, n/N (%) | 33 / 260 (12.7) | 21 / 159 (13.2) | 12 / 101 (11.9) | 1.13 | 0.54 - 2.47 |
| Intubation, n/N (%) | 193 / 260 (74.2) | 118 / 159 (74.2) | 75 / 101 (74.3) | 1.00 | 0.56 - 1.76 |
| Tracheotomy, n/N (%) | 245 / 260 (94.2) | 151 / 159 (95.0) | 94 / 101 (93.1) | 1.41 | 0.48 - 4.04 |
| Length of hospital treatment, days, median (IQR) | 11.0 (8.0) | 11.0 (9.0) | 11.0 (6.0) | 1.03 | 0.99 - 1.07 |
| Length of ICU treatment, days, median (IQR) | 5.0 (11.0) | 5.0 (11.0) | 5.0 (10.0) | 1.02 | 0.99 - 1.06 |
| Antihypertensive drugs at discharge |  |  |  |  |  |
| none, n/N (%) | 8 / 260 (3.1) | 2 / 159 (1.3) | 6 / 101 (5.9) |  |  |
| one, n/N (%) | 13 / 260 (5.0) | 6 / 159 (3.8) | 7 / 101 (6.9) | 2.57 | 0.40 - 22.40 |
| more than one, n/N (%) | 239 / 260 (91.9) | 151 / 159 (95.0) | 88 / 101 (87.1) | 5.15 | 1.16 - 35.66 |
| Secondary hypertension†, n/N (%) | 2 / 260 (0.8) | 2 / 159 (1.3) | 101 / 101 (100.0) | NA | NA - NA |
| Discharge to |  |  |  |  |  |
| rehabilitation, n/N (%) | 235 / 260 (90.4) | 146 / 159 (91.8) | 89 / 101 (88.1) | 1.51 | 0.65 - 3.48 |
| other, n/N (%) | 25 / 260 (9.6) | 13 / 159 (8.2) | 12 / 101 (11.9) |  |  |
| Disturbance cerebrospinal fluid flow, n/N (%) | 237 / 260 (91.2) | 140 / 159 (88.1) | 97 / 101 (96.0) | 0.30 | 0.09 - 0.84 |
| Intraventricular bleeding, n/N (%) | 78 / 260 (30.0) | 51 / 159 (32.1) | 27 / 101 (26.7) | 1.29 | 0.75 - 2.27 |
| Additional CT angiography, n/N (%) | 22 / 260 (8.5) | 8 / 159 (5.0) | 14 / 101 (13.9) | 0.33 | 0.13 - 0.80 |
| Additional MRI, n/N (%) | 128 / 260 (49.2) | 48 / 159 (30.2) | 80 / 101 (79.2) | 0.11 | 0.06 - 0.20 |
| Additional DSA, n/N (%) | 41 / 260 (15.8) | 7 / 159 (4.4) | 34 / 101 (33.7) | 0.09 | 0.04 - 0.20 |
| Cardiac rhythm |  |  |  |  |  |
| Sinus rhythm, n/N (%) | 238 / 260 (91.5) | 148 / 159 (93.1) | 90 / 101 (89.1) |  |  |
| atrial fibrillation, n/N (%) | 22 / 260 (8.5) | 11 / 159 (6.9) | 11 / 101 (10.9) | 0.61 | 0.25 - 1.48 |
| Left ventricular hypertrophy |  |  |  |  |  |
| any, n/N (%) | 156 / 260 (60) | 113 / 159 (71.1) | 43/101(42.6) | 3.31 | 1.97 - 5.62* |
| mild, n/N (%) | 107 / 260 (41.2) | 79 / 159 (49.7) | 28 / 101 (27.7) | 3.56 | 2.01 - 6.42 |
| moderate, n/N (%) | 35 / 260 (13.5) | 23 / 159 (14.5) | 12 / 101 (11.9) | 2.42 | 1.10 - 5.51 |
| severe, n/N (%) | 14 / 260 (5.4) | 11 / 159 (6.9) | 3 / 101 (3.0) | 4.62 | 1.35 - 21.33 |
| Left atrial enlargement, n/N (%) | 119 / 257 (46.3) | 82 / 157 (52.2) | 37 / 100 (37.0) | 1.86 | 1.12 - 3.13 |
| Diastolic dysfunction, n/N (%) | 214 / 260 (82.3) | 132 / 159 (83.0) | 82 / 101 (81.2) | 1.13 | 0.59 - 2.16 |
| Mitral valve regurgitation, n/N (%) | 139 / 260 (53.5) | 81 / 159 (50.9) | 58 / 101 (57.4) | 0.77 | 0.46 - 1.27 |
| Any mitral valve changes, n/N (%) | 33 / 259 (12.7) | 19 / 159 (11.9) | 14 / 100 (14.0) | 0.83 | 0.40 - 1.78 |
| Tricuspid valve regurgitation, n/N (%) | 117 / 259 (45.2) | 67 / 159 (42.1) | 50 / 100 (50.0) | 0.73 | 0.44 - 1.20 |
| Aortic valve regurgitation, n/N (%) | 65 / 259 (25.1) | 36 / 159 (22.6) | 29 / 100 (29.0) | 0.72 | 0.41 - 1.27 |
| Aortic valve stenosis, n/N (%) | 18 / 260 (6.9) | 8 / 159 ( 5.0) | 10 / 101 (9.9) | 0.48 | 0.18 - 1.27 |
| Any aortic valve changes, n/N (%) | 74 / 260 (28.5) | 37 / 159 (23.3) | 37 / 101 (36.6) | 0.52 | 0.30 - 0.91 |
| Right heart burden, n/N (%) | 22 / 259 (8.5) | 13 / 159 ( 8.2) | 9 / 100 (9.0) | 0.90 | 0.37 - 2.26 |
| Diameter left atrium, mm, median (IQR) | 40.0 (9.0) | 41.0 (8.0) | 38.0 (9.0) | 1.04 | 1.00 - 1.09 |
| Left ventricular end-diastolic diameter, mm, median (IQR) | 45.0 (7.0) | 46.0 (8.0) | 44.0 (7.0) | 1.02 | 0.98 - 1.06 |
| End diastolic interventricular septum wall thickness, mm, median (IQR) | 13.0 (3.0) | 13.0 (2.0) | 12.0 (2.0) | 1.35 | 1.17 - 1.58 |
| Restricted left ventricular function, n/N (%) | 19 / 259 (7.3) | 11 / 159 ( 6.9) | 8 / 100 (8.0) | 0.85 | 0.33 - 2.28 |
| Left ventricular ejection fraction, per cent, median (IQR) | 60.0 (0.0) | 60.0 (5.0) | 60.0 (0.0) | 0.99 | 0.95 - 1.04 |
| Right ventricular ejection fraction, per cent, median (IQR | 60.0 (5.0) | 60.0 (5.0) | 60.0 (0.0) | 1.00 | 0.95 - 1.07 |
| Wall motion abnormalities, n/N (%) | 16 / 259 (6.2) | 10 / 159 (6.3) | 6 / 100 (6.0) | 1.05 | 0.38 - 3.18 |
| Pericardial effusion†, n/N (%) | 10 / 258 (3.9) | 7 / 159 (4.4) | 3 / 99 (3.0) | 0.68 | 0.14 – 2.50 |
